# Developmental referrals of pre‐school children in a diverse community in England: The importance of parental migration for referral rates

**DOI:** 10.1111/cch.13009

**Published:** 2022-04-13

**Authors:** Tom Allport, Alissamaryam Ambrose, Simon M. Collin

**Affiliations:** ^1^ Centre for Academic Child Health, Bristol Medical School University of Bristol Bristol UK; ^2^ Community Children's Health Partnership Sirona CIC Bristol UK; ^3^ Brighton & Sussex University Hospitals NHS Trust Brighton UK; ^4^ Public Health England London UK

**Keywords:** autism, child development, ethnicity, migrant, referral

## Abstract

**Background:**

Children born to migrant parents have higher rates of language difficulties, intellectual disability and autism. This study explores the relationship between migration, ethnicity and reasons for early years referrals to community paediatrics in a diverse multi‐cultural population in a city in south west England.

**Methods:**

Observational retrospective study from a community paediatric service serving a multi‐cultural urban population from June 2012 to February 2016. We tested associations of ethnicity and parental birth origin with reason for referral (developmental or non‐developmental) for children under 5 years old and estimated crude rate ratios for referrals using population census data.

**Results:**

Data were available for 514 children (52% white or mixed race, 16% Asian, 21% African diaspora, and 11.5% Somali); 53% had two UK‐born parents while 22% had two migrant (non‐UK‐born) parents. Referrals were for developmental reasons in 307 (60%) including 86 for possible autism. Parental birth origin and ethnicity were associated with reason for referral (*p* < 0.001). Children from African diaspora, Asian or Somali backgrounds had more than twice the rate (rate ratio [RR] 2.37, 95% CI 1.88–2.99, *p* < 0.001) of developmental referrals compared with white or mixed‐race children. Children of Somali or African diaspora ethnicity were, respectively, six‐times (RR 5.99, 95% CI 3.24–10.8, *p* < 0.001) and four times (RR 4.23, 95% CI 2.44–7.29, *p* < 0.001) more likely to be referred for possible autism spectrum than their white or mixed‐race peers. Developmental referral as a proportion of all referrals was twice as high among children with one migrant parent (20.4%) and three times as high among children with two migrant parents (29.5%), compared with children whose parents were both UK‐born (10.7%).

**Conclusions:**

This study supports the importance of ethnicity and parental migration as factors in young children experiencing developmental difficulties, especially concerns about social communication or autism.

**Key messages**

- Pre‐school children from ethnically diverse and migrant backgrounds were referred more often with concerns about development than white British children
- Children of Somali or African diaspora ethnicity were 4–6 times more likely to be referred for concerns about autism than their white British peers
- Commissioners should prioritize targeted and tailored early years and environmental interventions to migrant families and communities.

## 1 INTRODUCTION

Migration to a different culture may affect early opportunities for play and social interaction, which are essential for the development of children's cognitive and social skills. Parental migration status, interacting with disadvantage, is generally associated with worse early learning outcomes for children born in the host country (Allport et al., 2019; Fazel & Betancourt, 2018). Children born to migrant parents often have higher rates of language delay (Hoff, 2013) and are at greater risk of autistic and neurodevelopmental disorders (Gardener et al., 2009; Magnusson et al., 2012). Black children in the United States had a higher risk of autism than white children that was amplified if the mother was foreign‐born (Becerra et al., 2014), while a UK study found that increased risk of autism‐spectrum disorders in black and Asian children was attributable to much higher incidence among children of first‐generation immigrant parents (Keen et al., 2010).

Early years interventions are highly effective and cost‐effective in preventing mental health difficulties and educational failure by reducing pre‐ and post‐natal exposure to biological and environmental stresses (Shonkoff & Fisher, 2013; Strelitz et al., 2013). However, early years interventions may not reach the children of refugees and disadvantaged migrants (Tyrer & Fazel, 2014). Targeting and tailoring interventions help them be effective where they are most needed (Marmot et al., 2010), but this ‘proportionate universalism’ requires an understanding of the developmental issues more likely to be encountered in migrant families.

Somalis fleeing political instability and violence constitute one of the largest diasporas in the world (Hammond et al., 2011). Many immigrant Somali families face challenges including poverty, insecurity, unemployment, isolation and discrimination (Osman et al., 2016). The city of Bristol in the south west of England has a large multicultural population, and 5% of children in the city have Somali parents. These children are educationally underachieving at all key stages compared to their non‐Somali peers, and this gap appears to widen at ages 7–11 and 15–16 years (Bristol City Council, 2014). Risks of developmental and non‐developmental delay in children born recently into this specific group in the United Kingdom relative to UK‐born and other ethnic groups have not be described. In this study, we used community paediatric service data from a 4‐year period to investigate the relationship between specific ethnic groups and referral of pre‐school children for developmental or non‐developmental reasons. We also investigated the association of parental and child birth origin, that is, migration in general, with reason for referral. Our hypothesis, based on anecdotal evidence from our service and evidence from studies in other settings, was that children from black and minority ethnic groups and of non‐UK‐born parents would be represented disproportionately among referrals for both developmental and non‐developmental reasons. Our underlying aim was to use evidence from our study, if obtained, to advocate for interventions within children's services prioritizing the groups at highest risk of developmental issues.

## 2 METHODS

### 2.1 Study design

This was an observational retrospective study using routinely collected data from a community paediatric service in the city of Bristol, in south west England (UK).

### 2.2 Study population and setting

Bristol has a population of 463,000 of which 85,800 are under the age of 16 years and an estimated 16% are black, Asian and minority ethnic (BAME) comprising black 6%, Asian 6% and mixed race/dual heritage 4%. East and central Bristol is the most diverse part of the city, with 50% of 9500 children under 5 years children having a BAME background (Bristol City Council & Bristol Clinical Commissioning Group, 2014). These areas of the city are served by a multi‐disciplinary team of speech and language therapists, occupational therapists, physiotherapists, clinical psychologists, Somali link workers/therapy assistants and community paediatricians who have expertise in the management of children with long‐term health problems which may have an impact on other areas of their lives. Assessment and treatment are provided for children with developmental problems such as delayed milestones, learning disabilities, autism, cerebral palsy and attention deficit hyperactivity disorder (ADHD). Paediatricians also review the health needs of children in care, support vulnerable children and their families, work with education and safeguarding teams and lead investigations into unexpected child deaths.

Referrals to the team are made from primary (health visitors and general practitioners) and secondary care (neonatal and general paediatric teams) and from educational and social care (special educational needs and other specialist teaching/education staff and social workers). The team operates jointly with a Child and Adolescent Mental Health Service (CAMHS) and a Barnardo's HYPE (Helping Young People to Engage) charitable service as the Community Children's Health Partnership for Bristol and the neighbouring counties of South Gloucestershire and North Somerset, under the management of Sirona Care and Health Community Interest Company. The service is commissioned for the local population under a ‘block contract’ by NHS Bristol, North Somerset and South Gloucestershire Clinical Commissioning Group (CCG).

Our study was based on retrospective review of (paper) case files to determine the reason for referral, ethnicity and parental birth origin of children seen by our team over a 4‐year period. Inclusion criteria were all patients who attended the health partnership between June 2012 and February 2016 and who were under or at the age of 5 years during their first appointment. June 2012 was selected as the start date because this is when ethnicity began to be routinely captured. All files held within the community paediatric office at the time of the review (February 2016) were examined. Closed case files could not be examined because these are routinely transferred to off‐site storage.

### 2.3 Data collection and case definition

Case files were examined to extract reason for referral (developmental or non‐developmental), ethnicity and parental birth origin. A developmental referral was classified if the reason for referral included one or more of the following issues/questions: possible speech and language delay or disorder; difficulties with social interaction or possible autism spectrum disorder (ASD); developmental delay/disorder or possible learning difficulty; difficulties with motor function or possible cerebral palsy; concern about sensory functioning; possible ADHD; and possible/confirmed genetic disorder, for example, Down Syndrome. Referrals not including these questions were classified as non‐developmental, and developmental referrals were categorized as ASD or non‐ASD. Ethnicity of the child was categorized using the UK 2001 Level 2 Census classification (Office for National Statistics, 2001), with the additional category Somali collected by Bristol City agencies. Parental birth origin was split into categories: two migrant (any non‐UK country of origin) parents; one migrant parent; two UK‐born parents; child born abroad.

### 2.4 Data analysis

Associations of ethnicity and parental birth origin (exposures) with type of referral (outcome) were tested using Chi‐squared tests. Crude referral rate ratios with 95% confidence intervals were estimated from referral rates for the study period using counts of children in each exposure and outcome group as numerators and population census data for BAME under 5 years old in East and Central Bristol as denominators (Bristol City Council & Bristol Clinical Commissioning Group, 2015; Office for National Statistics, 2014). All analysis was performed using Stata (StataCorp. 2017. *Statistical Software: Release 15*. College Station, TX: StataCorp LLC).

## 3 RESULTS

During the study period there were 1246 referrals, of which case data were extracted for 514 children (41.3%). Of these, 58% (*n* = 296) were male, with a mean age of 3.5 years. Ethnicity was 42.2% (*n* = 217) white British, 7.0% (*n* = 36) white other, 10.1% (*n* = 52) Caribbean, 10.7% (*n* = 55) African, 15.9% (*n* = 82) Asian, 2.5% (*n* = 13) mixed other and 11.5% (*n* = 59) Somali; 52.7% (*n* = 271) of cases had two UK‐born parents while 21.8% (*n* = 112) had two migrant parents (Table 1, Table 2).

**TABLE 1:** Developmental and non‐developmental referrals of children age under 5 years from June 2012 to February 2016 in central and east Bristol by birth origin of parents

Parental birth origin	Autism spectrum disorder (ASD)	Non‐ASD developmental	Non‐developmental	Total
Both migrant	33 (29.5%)	59 (52.7%)	20 (17.8%)	112 (100.0%)
One migrant	20 (20.4%)	48 (49.0%)	30 (30.6%)	98 (100.0%)
Child born abroad	4 (12.1%)	18 (54.6%)	11 (33.3%)	33 (100.0%)
Both UK‐born	29 (10.7%)	96 (35.4%)	146 (53.9%)	271 (100.0%)
Total	86 (16.7%)	221 (43.0%)	207 (40.3%)	514 (100.0%)

**TABLE 2:** Developmental and non‐developmental referrals of children age under 5 years from June 2012 to February 2016 in central and east Bristol by ethnicity of child

Ethnicity of child	Autism spectrum disorder (ASD)	Non‐ASD developmental	Non‐developmental	Total
White or mixed	32 (12.0%)	110 (41.4%)	127 (46.6%)	266 (100.0%)
Asian	7 (8.5%)	46 (56.1%)	29 (35.4%)	82 (100.0%)
African diaspora	27 (25.2%)	33 (30.8%)	47 (43.9%)	107 (100.0%)
Somali	20 (33.9%)	32 (54.2%)	7 (11.9%)	59 (100.0%)
Total	86 (16.7%)	221 (43.0%)	207 (40.3%)	514 (100.0%)

Referrals were due to developmental concern in 59.7% (*n* = 307) of cases. Reasons for non‐developmental referral included vulnerable or looked‐after children (6.0%, *n* = 31), heart disease (1.6%, *n* = 8), feeding or weight concerns (6.4%, *n* = 33) and other (including issues such as toileting, skin and hearing).

**FIGURE 1:**
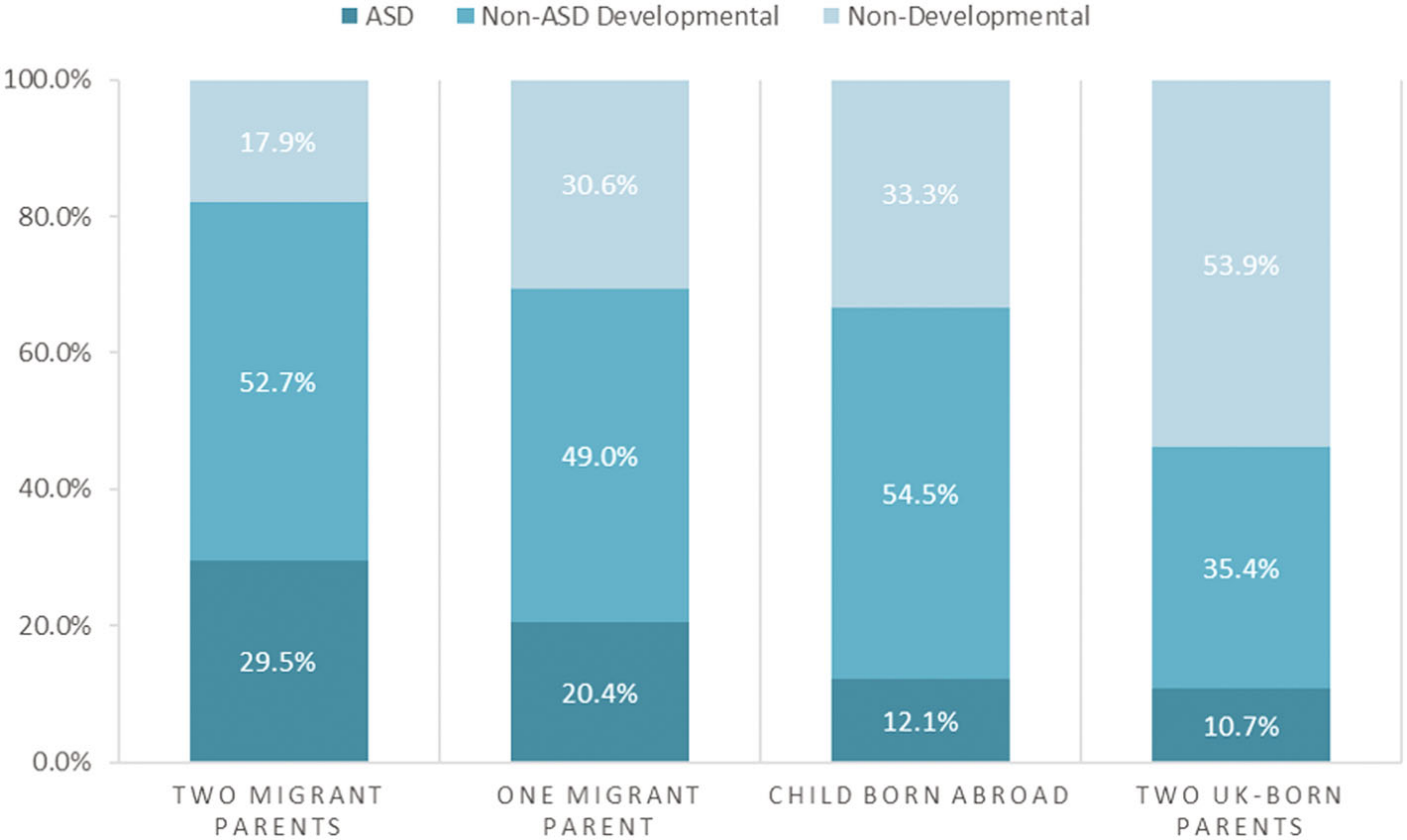
Reason for referral of children age under 5 years from June 2012 to February 2016 in central and east Bristol by parental origin

**FIGURE 2:**
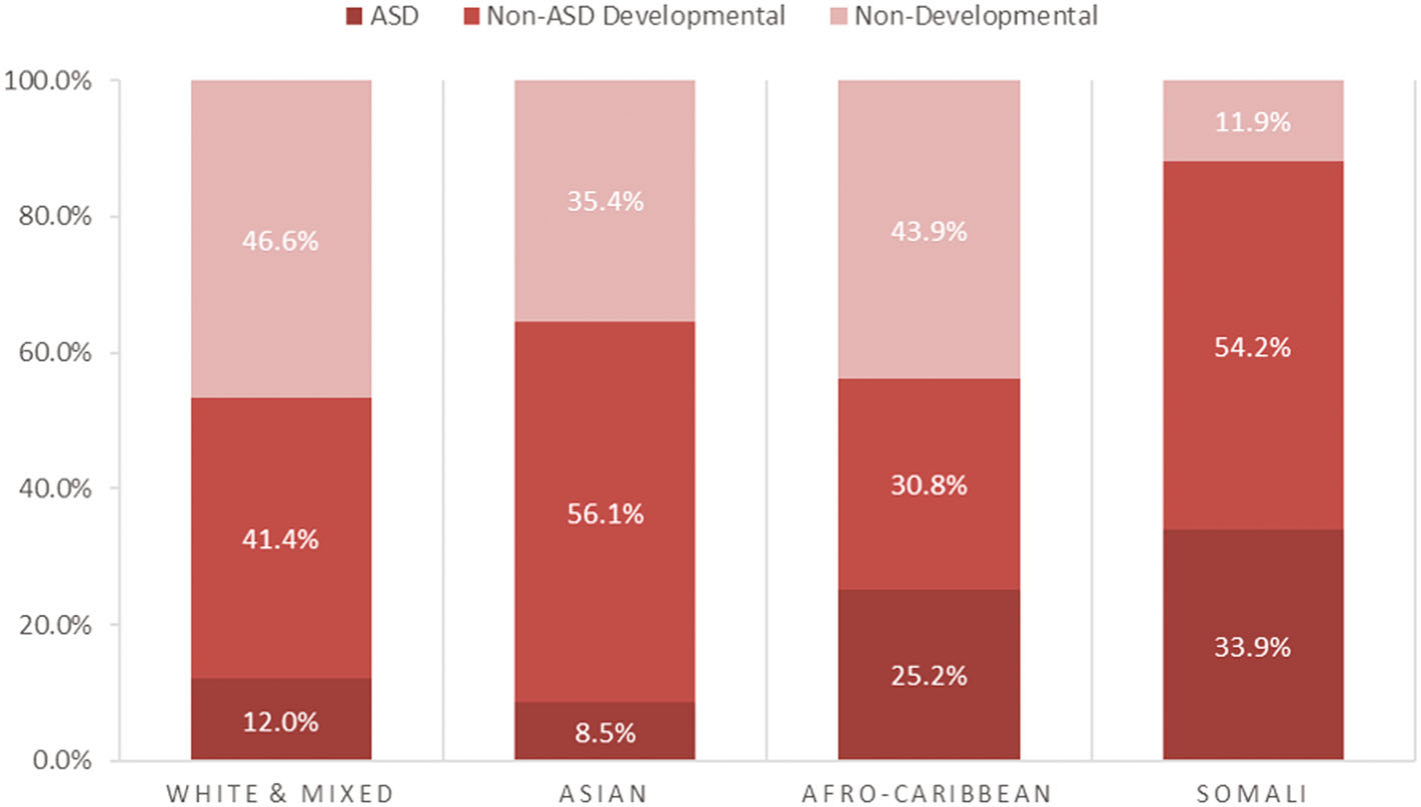
Reason for referral of children age under 5 years from June 2012 to February 2016 in central and east Bristol by child's ethnicity

**FIGURE 3:**
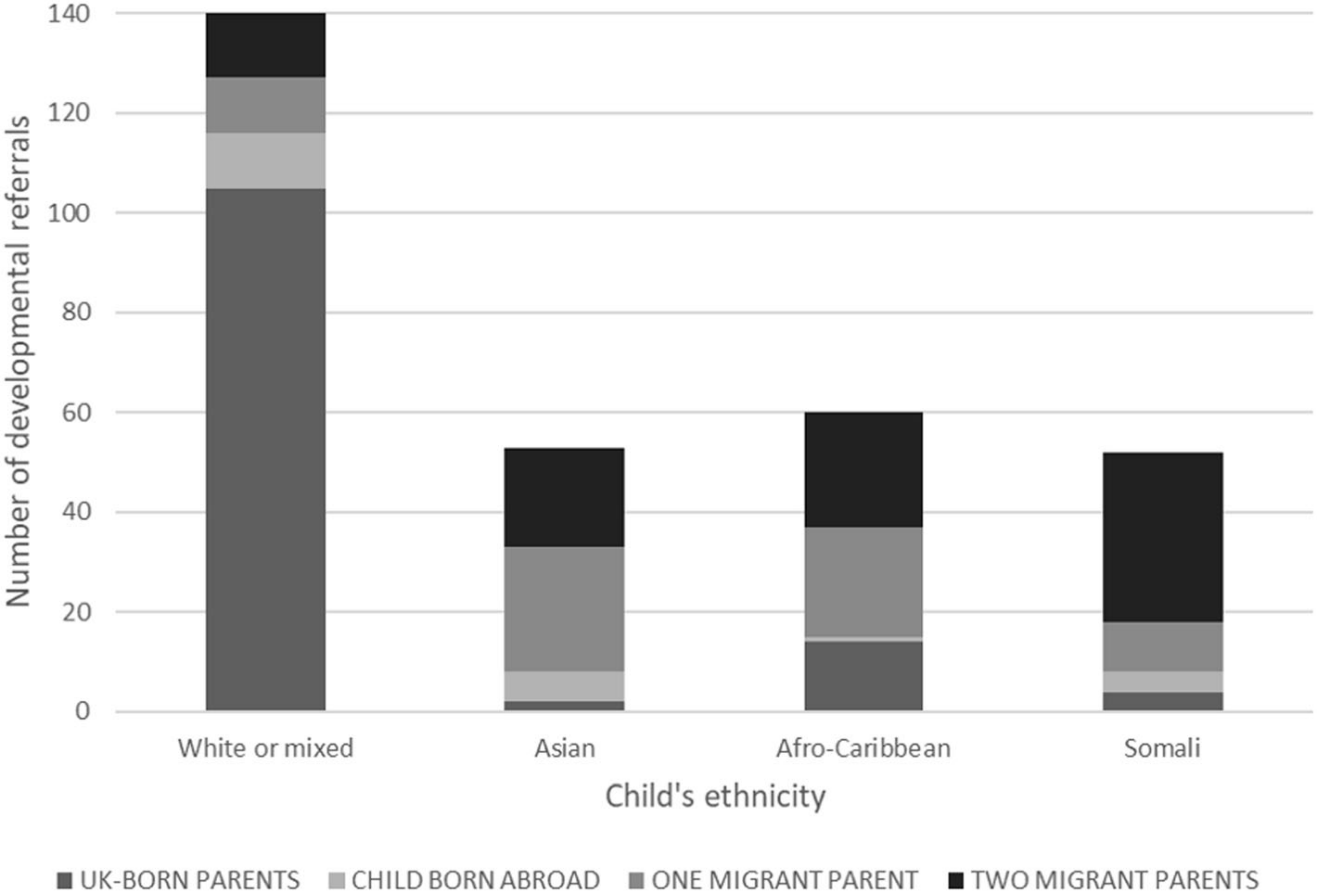
Number of developmental referrals of children age under 5 years from June 2012 to February 2016 in central and east Bristol by child's ethnicity and parental origin

Parental birth origin (Table 1 and Figure 1) and child's ethnicity (Table 2 and Figure 2) were strongly associated with developmental reason for referral (Chi‐squared *p* values both <0.001). Developmental referrals by ethnicity and parental birth origin, are presented in Figure 3 (Table S1 for data). Developmental referral as a proportion of all referrals was twice as high among children with one migrant parent (20.4%) and three times as high among children with two migrant parents (29.5%), compared with children whose parents were both UK‐born (10.7%) (Table 1); the proportion of developmental referrals was twice as high among children of African diaspora ethnicity (25.2%) and three times as high among children of Somali ethnicity (33.9%) compared with children of white or mixed ethnicity (12.0%) (Table 2).

Children from black, Asian and minority ethnic backgrounds had more than twice the rate (rate ratio [RR] 2.37, 95% CI 1.88–2.99, *p* < 0.001) of developmental referrals compared with white or mixed‐race children (Table 3). Children of Somali ethnicity were six times more likely (RR 5.99, 95% CI 3.24–10.8, *p* < 0.001), and those with other African or Caribbean ethnicity four times more likely (RR 4.23, 95% CI 2.44–7.29, *p* < 0.001) to have been referred for ASD, than their local white or mixed heritage British peers (Tables S2 and S3 for data).

**TABLE 3:** Rate ratios (RR) for developmental and non‐developmental referrals of children age under 5 years from June 2012 to February 2016 in central and east Bristol by ethnicity of child (see Tables S2 and S3 for data)

Ethnicity of child	East and central Bristol under‐5 population	Autism spectrum disorder (ASD)	Non‐ASD developmental	Developmental (ASD and non‐ASD)	Non‐developmental	Developmental and non‐developmental
		RR (95% CI)	RR (95% CI)	RR (95% CI)	RR (95% CI)	RR (95% CI)
White or mixed	6130	1.00 (reference)	1.00 (reference)	1.00 (reference)	1.00 (reference)	1.00 (reference)
Asian	1142	1.17 (0.44, 2.71)	2.24 (1.56, 3.19)	2.00 (1.43, 2.76)	1.26 (0.81, 1.89)	1.78 (1.45, 2.16)
African diaspora	1223	4.23 (2.44, 7.29)	1.50 (0.99, 2.24)	2.12 (1.54, 2.88)	1.90 (1.33, 2.68)	2.05 (1.70, 2.46)
Somali	640	5.99 (3.24, 10.8)	2.79 (1.82, 4.16)	3.51 (2.50, 4.85)	0.54 (0.21, 1.15)	2.61 (2.09, 3.22)

## 4 DISCUSSION

This study has identified differential referral patterns for early developmental assessment in children from a UK urban population according to ethnicity and parental migration status, with much higher rates of referral for possible autism in children with parents of Somali or African diaspora origin and higher rates of all developmental referrals among children of Asian and African ethnicity. Three in five children of Somali ethnicity had two first generation migrant parents, reflecting the more recently established Somali community in Bristol, but 80% of Asian and 60% of referred African diaspora children had at least one first generation migrant parent despite the long‐established Caribbean and South Asian populations in Bristol. Even within African diaspora and South Asian populations with longstanding presence in Bristol, most children referred for developmental reasons including possible autism had one or two migrant parents, suggesting migration may be more important than ethnicity as a background to developmental difficulties. Our sample size was not large enough to support analyses stratified by migrant status and parental ethnicities, and we did not have the population denominators required to estimate relative referral rates by parental birth origin, but our findings mirror those of a study in London which found that higher risks of ASD in black and Asian children were driven by much higher incidence among children of first‐generation immigrant parents (Keen et al., 2010). As in our study, the relative risks of ASD in the London study were also substantial, 8.2‐fold and 5.5‐fold higher for children of black and Asian immigrant mothers (living in Lambeth), respectively, compared to children of white UK‐born mothers.

The community paediatric service from which we obtained our data is the only provider of specialist care for children with long‐term health problems in the study area; therefore, our study population is likely to be representative of the overall population. We cannot say with certainty whether our findings are generalizable to other settings, but other cities in the United Kingdom have broadly similar service providers, referral systems and ethnically mixed and migrant populations. We note that a Swedish study found substantially higher (2 to 3.5‐fold) odds of low‐functioning ASD, that is, with intellectual disability (but lower odds of high‐functioning autism) among children of migrant parents from east Africa (Somalia and Ethiopia) and other sub‐Saharan African countries compared with children of parents born in Sweden (Magnusson et al., 2012), suggesting a mechanism by which children of parents from these regions of origin are more likely to be diagnosed with severe ASD in any well‐resourced health system. Indeed, the Swedish study found that the risk of low‐functioning autism was highest when migration occurred in the year before birth, suggesting a causal mechanism rather than an effect of ethnicity per se or bias related to case ascertainment (Magnusson et al., 2012). Further research is required to investigate the mechanisms of this important observation.

Conversely, the lower odds of high‐functioning ASD were attributed by the Swedish authors to under‐diagnosis possibly related to detecting defects in social interaction within different cultural milieu (Magnusson et al., 2012). Given that we found consistently and substantially higher rates of referrals across ethnic groups, we think it unlikely that these effects could be attributed to culturally related differences in access to health services, recognition of developmental delay, and/or referral decision‐making between recent migrants and the more established UK population. Instead, these are aspects of health provision which typically need to be improved for minority ethnic and migrant groups (Suphanchaimat et al., 2015), along with broader consideration of how host countries welcome and support families migrating from cultures where ‘it takes a village to raise a child’ to counteract an increasingly hostile environment for migrants in the United Kingdom (Allport et al., 2019). At the local level, culturally sensitive knowledge of how conditions such as autism are viewed in minority ethnic communities is essential to reducing stigma and providing support to encourage families not to delay seeking help for their children (Fox et al., 2017).

### 4.1 Implications for policy and practice

Early child development interventions are effective and highly cost‐effective for youth and adult outcomes (Knudsen et al., 2006). Bristol's experience of suboptimal educational outcomes for children and adolescents of Somali and other migrant origin is comparable to those observed in other UK cities (Demie, 2015, 2018; Sporton & Valentine, 2007; Strand et al., 2015; Welford & Montague, 2017). Somalis, and professionals they work with, share concerns about poor youth outcomes (Betancourt et al., 2017; Bhui et al., 2006; Ellis et al., 2020; Gillespie et al., 2020; Hodes & Vostanis, 2019; Warfa et al., 2006). Our findings point to the potential long‐term benefits of intervention in early childhood for refugee and disadvantaged migrant populations.

We have urged commissioners of children's services in England and Wales to prioritize targeted and tailored early years and environmental interventions to migrant families and communities (Allport et al., 2021), consistent with the concept of ‘proportionate universalism’ which was first proposed to address health inequalities in the United Kingdom (Marmot et al., 2010). Our finding that developmental issues disproportionately affect pre‐school children in migrant families adds to evidence that these families need special attention. Early years interventions should develop or reinforce community assets, support networks and communal child‐rearing practices, by supporting community groups and activities to help families ‘Find your village’, help families navigate unfamiliar systems (e.g., housing, benefits and family reunion entitlements), improve access to universal early years services (e.g., children's centres) and English language classes and provide accessible public health messages (importance of play, communication, interaction and restricting TV/screen use) in community languages and via community groups. These goals are jointly the concern of early years teams working in health and education, so further alignment and integration might allow many of these changes without substantial cost. Many of these interventions could also be facilitated by community link workers, for example, under the umbrella of Social Prescribing (Gheera & Eaton, 2020). We also urge Housing, Planning and Neighbourhood Services to provide or enable communal space in local authority housing for families to meet others, facilitate local street closures for outdoor play and improve parks and green spaces by ensuring child‐safe fencing and gates, providing sufficient equipment, zoned by age, to sustain children's attention, and adjacent seating so that parents/carers can meet, and still see their children play, and providing youth‐friendly areas alongside areas for younger children.

### 4.2 Strengths and limitations

Referral is only a proxy for diagnosis, and we did not have data to determine how good a proxy referral was as an outcome measure. However, referral can be considered to indicate concern about social communication and interaction needs, which are likely to represent persistent developmental and educational challenges. Our referral rate ratios were not adjusted for potential confounding factors such as parental age, parental education, household socio‐economic status, family size and child's birth weight, gestational age, gender, birth order and exact age (Gardener et al., 2009; Keen et al., 2010). Adjustment for parental age and family disposable income only moderately attenuated crude associations of maternal birth origin with odds of ASD in a Swedish study (Magnusson et al., 2012). Given the substantial rate ratios that we observed in our study, we might reasonably expect significant effects to remain had we been able to adjust for confounders. However, the role of confounders in explaining (or not) differences in child development by ethnicity is a limitation of our study which would need to be addressed in any follow‐up study and to provide stronger evidence in support of policy recommendations (Kelly et al., 2006).

Non‐attendances following referral were not included in the study, which may have underrepresented ethnically diverse children if they were less likely to access the service. A substantial proportion of case files were closed and unavailable for data extraction. This could have led to bias if case closure was differential across ethnicities, which we cannot determine without examining all closed cases. If ethnically diverse children tend to have a longer lead‐time from referral to autism spectrum diagnosis than their white peers (Martin‐Herz et al., 2012), because of professional uncertainty about the cross‐cultural appropriateness of diagnostic processes and instruments and parental reluctance to accept diagnoses (Daley, 2016; Norbury & Sparks, 2013), analysing open case files in the under 5 age group could mean that BAME children are over‐represented; conversely, if they are more likely to be referred at an older age, our study could under‐represent rates in these populations. Any factors unrelated to child development which led to case files for children of minority ethnic or migrant parents being open for longer could induce an apparent association between these exposures and referral, but the magnitude of the observed effects suggests that these factors would be clearly discernible. Our local primary care teams have longstanding ‘*Healthlinks*’ interpreting and advocacy services which promote inclusive practice and our service has Somali Link Workers who are not involved in contact with families prior to referral. We do not believe these services significantly influenced the observed associations.

## 5 CONCLUSIONS

In this study, Somali children born in Bristol appeared six times more likely, and African diaspora children four times as likely, to be referred for consideration of the possibility of autism. Our findings require replication and more in‐depth exploration, including collection of data on other factors possibly related to developmental problems and referrals. In the meantime, we urge commissioners to consider prioritizing targeted and tailored early years interventions to migrant families and communities.

## Supporting information


**Table S1:** Developmental and non‐developmental referrals from June 2012 to February 2016 in central and east Bristol by ethnicity of child and migratory status of parents

**Table S2:** Numerators (cases) and population denominators for developmental and non‐developmental referrals from June 2012 to February 2016 in central and east Bristol by ethnicity of child

**Table S3:** Referral rates from June 2012 to February 2016 per 100 children <5 years old in central and east Bristol for developmental and non‐developmental referrals by ethnicity of child (calculated from data shown in Table S2)

## Data Availability

The data that support the findings of this study are available from the corresponding author upon reasonable request.
