# Supplementary material for: Developmental referrals of pre‐school children in a diverse community in England: The importance of parental migration for referral rates
Source: Child Care Health Dev. 2022 Apr 13;49(2):240–7. doi: 10.1111/cch.13009 (PMC10084135; doi:10.1111/cch.13009)
Supplement: Supplementary file 1 — Table S1: Developmental and non‐developmental referrals from June 2012 to February 2016 in central and east Bristol by ethnicity of child and migratory status of parents [file CCH-49-240-s003.docx]

**Table S1: Developmental and non-developmental referrals from June 2012 to February 2016 in central and east Bristol by ethnicity of child and migratory status of parents**

| Ethnicity of child and parental origin | Autism spectrum disorder (ASD) | Non-ASD developmental | Non-developmental | Total |
| --- | --- | --- | --- | --- |
| White or mixed | n=32 | n=110 | n=127 | n=266 |
| Both migrant | 5 (15.6%) | 10 (9.1%) | 4 (3.2%) | 19 (7.1%) |
| One migrant | 2 (6.3%) | 9 (8.2%) | 2 (1.6%) | 13 (4.9%) |
| Child born abroad | 3 (9.4%) | 8 (7.3%) | 5 (4.0%) | 16 (6.0%) |
| Both UK-born | 22 (68.8%) | 83 (75.5%) | 113 (91.1%) | 218 (82.0%) |
|  |  |  |  |  |
| Asian | n=7 | n=46 | n=29 | n=82 |
| Both migrant | 4 (57.1%) | 16 (34.8%) | 10 (34.5%) | 30 (36.6%) |
| One migrant | 3 (42.9%) | 22 (47.8%) | 10 (34.5%) | 35 (42.7%) |
| Child born abroad | 0 (0.0%) | 6 (13.0%) | 3 (10.3%) | 9 (11.0%) |
| Both UK-born | 0 (0.0%) | 2 (4.4%) | 6 (20.7%) | 8 (9.8%) |
|  |  |  |  |  |
| African diaspora | n=27 | n=33 | n=47 | n=107 |
| Both migrant | 11 (40.7%) | 12 (36.4%) | 4 (8.5%) | 27 (25.2%) |
| One migrant | 10 (37.0%) | 12 (36.4%) | 16 (34.0%) | 38 (35.5%) |
| Child born abroad | 0 (0.0%) | 1 (3.0%) | 2 (4.3%) | 3 (2.8%) |
| Both UK-born | 6 (22.2%) | 8 (24.2%) | 25 (53.2%) | 39 (36.5%) |
|  |  |  |  |  |
| Somali | n=20 | n=32 | n=7 | n=59 |
| Both migrant | 13 (65.0%) | 21 (65.6%) | 2 (28.6%) | 36 (61.0%) |
| One migrant | 5 (25.0%) | 5 (15.6%) | 2 (28.6%) | 12 (20.3%) |
| Child born abroad | 1 (5.0%) | 3 (9.4%) | 1 (14.3%) | 5 (8.5%) |
| Both UK-born | 1 (5.0%) | 3 (9.4%) | 2 (28.6%) | 6 (10.2%) |
