# Supplementary material for: Developmental referrals of pre‐school children in a diverse community in England: The importance of parental migration for referral rates
Source: Child Care Health Dev. 2022 Apr 13;49(2):240–7. doi: 10.1111/cch.13009 (PMC10084135; doi:10.1111/cch.13009)
Supplement: Supplementary file 2 — Table S2: Numerators (cases) and population denominators for developmental and non‐developmental referrals from June 2012 to February 2016 in central and east Bristol by ethnicity of child [file CCH-49-240-s001.docx]

**Table S2: Numerators (cases) and population denominators for developmental and non-developmental referrals from June 2012 to February 2016 in central and east Bristol by ethnicity of child**

| Ethnicity of child | Autism spectrum disorder (ASD) | Non-ASD Developmental | Developmental (ASD and non-ASD) | Non-Developmental | Developmental and non-developmental | East & central Bristol <5 population (denominator) |
| --- | --- | --- | --- | --- | --- | --- |
| White or mixed | 32 | 110 | 142 | 124 | 408 | 6130 |
| Asian | 7 | 46 | 53 | 29 | 135 | 1142 |
| African diaspora | 27 | 33 | 60 | 47 | 167 | 1223 |
| Somali | 20 | 32 | 52 | 7 | 111 | 640 |
