# Supplementary material for: Developmental referrals of pre‐school children in a diverse community in England: The importance of parental migration for referral rates
Source: Child Care Health Dev. 2022 Apr 13;49(2):240–7. doi: 10.1111/cch.13009 (PMC10084135; doi:10.1111/cch.13009)
Supplement: Supplementary file 3 — Table S3: Referral rates from June 2012 to February 2016 per 100 children <5 years old in central and east Bristol for developmental and non‐developmental referrals by ethnicity of child (calculated from data shown in Table S2) [file CCH-49-240-s002.docx]

**Table S3: Referral rates from June 2012 to February 2016 per 100 children <5 years old in central and east Bristol for developmental and non-developmental referrals by ethnicity of child (calculated from data shown in Table S2)**

| Ethnicity of child | ASD referral rate (95% CI) | Non-ASD referral rate (95% CI) | Developmental referral rate (95% CI) | Non-developmental referral rate (95% CI) | Developmental and non-developmental referral rate (95% CI) |
| --- | --- | --- | --- | --- | --- |
| White or mixed | 0.52 (0.36, 0.74) | 1.79 (1.47, 2.16) | 2.32 (1.95, 2.73) | 2.02 (1.68, 2.41) | 6.66 (6.03, 7.33) |
| Asian | 0.61 (0.25, 1.26) | 4.03 (2.95, 5.37) | 4.64 (3.48, 6.07) | 2.54 (1.70, 3.65) | 11.8 (9.91, 14.0) |
| African diaspora | 2.21 (1.45, 3.21) | 2.70 (1.86, 3.79) | 4.91 (3.74, 6.31) | 3.84 (2.82, 5.11) | 13.7 (11.7, 15.9) |
| Somali | 3.13 (1.91, 4.83) | 5.00 (3.42, 7.06) | 8.13 (6.07, 10.7) | 1.09 (0.44, 2.25) | 17.3 (14.3, 20.9) |
